# Comparison of long GnRH agonist versus GnRH antagonist protocol in poor responders

**DOI:** 10.4274/tjod.80090

**Published:** 2014-12-15

**Authors:** Sadık Şahin, Selçuk Selçuk, Belgin Devranoğlu, Tayfun Kutlu, Melda Kuyucu, Mustafa Eroğlu

**Affiliations:** 1 Zeynep Kamil Women and Children’s Diseases Education and Research Hospital, Clinics of Obstetrics and Gynecology, İstanbul, Turkey

**Keywords:** Poor ovarian response, long GnRH agonist, GnRH antagonist, IVF

## Abstract

**Objective:**

To compare long GnRH agonist with GnRH antagonist protocol in poor responders.

**Materials and Methods:**

Medical charts of 531 poor responder women undergoing in-vitro fertilization (IVF) cycle at Zeynep Kamil Maternity and Children’s Hospital, IVF Center were retrospectively analysed. Those who received at least 300 IU/daily gonadotropin and had ≤3 oocytes retrieved were enrolled in the study. Poor responders were categorized into two groups as those who received long GnRH agonist or GnRH antagonist regimen.

**Results:**

Treatment duration and total gonadotropin dosage were significantly higher in women undergoing the long GnRH agonist regimen compared with the GnRH antagonist regimen (p<0.001 for both). Although the number of total and mature oocytes retrieved was similar between the groups, good quality embryos were found to be higher in the GnRH antagonist regimen. The day of embryo transfer and number of transferred embryos were similar in the groups. No statistically significant differences were detected in pregnancy (10.5% vs 14.1%), clinical pregnancy (7.7% vs 10.6%) and early pregnancy loss rates (27.2% vs 35%) between the groups.

**Conclusion:**

GnRH antagonist regimen may be preferable to long GnRH regimen as it could decrease the cost and treatment duration in poor responders.

## INTRODUCTION

Poor ovarian response (POR) is one of the most challenging situations in assisted reproductive technology (ART), with disappointing overall in-vitro fertilization (IVF) success rates.

POR to ovarian stimulation usually indicates a reduction in follicular response, resulting in high cancellation rates or a reduced number of retrieved oocytes in women undergoing IVF^(1)^. In comparison to normal responders, these patients have impaired fertilization rates, lower embryo quality and decreased pregnancy rates^(2)^. Various treatment modalities have been proposed in an effort to improve ovarian response and IVF outcome. These include different regimens for pituitary suppression, the use of high doses of gonadotrophins, the use of gonadotropins with luteinizing hormone activity as well as adjuvant therapies^(3)^. The introduction of GnRH agonists (GnRHa) in assisted reproduction has increased the success rate of IVF treatment by reducing the incidence of a premature LH surge^(4)^. However, gonadotropin releasing hormone antagonists (GnRH-ant) have some advantages in mechanisms of actions over GnRHa. Unlike GnRHa, GnRH-ant act by the mechanism of competitive binding on GnRH receptors in pituitary and rapid action for pituitary suppression makes them rational to use in poor responders undergoing IVF^(5)^. However, most of these treatment regimens failed to increase the success rate of assisted reproduction in poor responders. The lack of uniform definition of POR is another confusing factor in previously reported studies, which makes it difficult to compare different regimens in these women. In an effort to make a universal definition of POR, a consensus was reached on the minimal criteria needed to define this issue by ESHRE working group in Bologna^(6)^. According to Bologna consensus, at least two of the following three features must be present:

1. Advanced maternal age (≥40 years) or any other risk factor for POR;

2. A previous POR (≤3 oocytes with a conventional stimulation protocol);

3. An abnormal ovarian reserve test (i.e. Antral follicle count <5-7 follicles or Antimullerian hormone <0.5-1.1 ng/ml). Additionally, two episodes of POR after maximal stimulation are sufficient to define a patient as poor responder in the absence of advanced maternal age or abnormal ovarian reserve test. Thus, the definition of POR by ESHRE group makes future meta-analysis more reliable and also prospective randomized trials more homogeneous.

The aim of this retrospective cohort study was to compare IVF outcome of poor responders undergoing the long GnRHa versus GnRH antagonist regimen.

## MATERIALS AND METHODS

This study was conducted as a retrospective cohort analysis of poor responder women who underwent IVF treatment with the long GnRHa or GnRH-ant protocol at Zeynep Kamil Maternity and Children’s Hospital IVF center between January 2008 to April 2014. The medical charts of women who had ≤3 oocytes retrieved were reviewed and their demographic characteristics and IVF data were recorded. Mild stimulation cycles, the cycles obtained >3 oocytes and cycles stimulated with <300 IU/day gonadotropins were excluded from the study. The local Ethics Committee approved the study.

In the long GnRHa regimen, pituitary down-regulation with either a subcutaneous injection of triptorelin 0.1 mg daily (Decapeptyl 0.1 mg, Ferring) or leuprolide acetate 10 IU daily (Lucrin 5mg/ml, Abbvie) was commenced in the midluteal phase of the menstrual cycle and continued until the second day of menstrual cycle. This was followed by an ultrasound confirmation of down-regulation by endometrium thickness and measurement of estradiol. When down-regulation was achieved, ovarian stimulation was commenced with gonadotropin injections (Recombinant FSH, hMG or urinary follitropin) at a dose of 300-450 IU/day and continued with a half of applied dose of triptorelin (0.05 mg/daily) or leuprolide acetate (5 IU/daily) until the administration of hCG injection. In the GnRH-ant regimen, gonadotropin injections at a dose of 300-450 IU/day were commenced on day 2 or 3 of the cycle. Antagonists (Cetrorelix 0.25 mg, Merck Serono) were added to stimulation protocol when the leading follicle achieved ≥13 mm in diameter. In both groups, the hCG injection (Ovitrelle 6.500 IU/day; Merck-Serono) was administered aiming at least one follicle reaching ≥17 mm in diameter to trigger final oocyte maturation. The luteal phase support in both groups was provided with the daily administration of 600 mg of progesterone intravaginally (Progestan 200 mg cap., Kocak Farma) or 90 mg of progesterone gel intravaginally (Crinone 8% gel, Merck Serono) until clinical pregnancy was achieved.

Pregnancy was defined as a positive β-hCG measurement 12 days following embryo transfer. Clinical pregnancy was diagnosed when fetal heart motion was observed during sonographic examination. Early pregnancy loss rate was defined as pregnancy losses that occurred in the time interval between a positive hCG test and the appearance of fetal heart beats. Good-quality embryos were defined as embryos with a normal cleavage rate and 10% fragmentation.

Statistical analyses were performed using the SPSS 17.0 program (Inc., Chicago, IL, USA) and R statistical software. Continuous variables were presented as mean ± standard deviation (SD) and categorical variables were defined as percentages (%). Continuous variables were compared with the Student’s t-test, while chi-squared or Fisher’s exact tests were used for comparison of categorical variables. P value <0.05 represents significance.

## RESULTS

A total of 531 women who reached the oocyte-pick up procedure were recruited in this study. Of these women, 311 underwent with the long GnRHa regimen and 220 underwent with the GnRH-ant regimen. Demographic and clinical data of patients are presented in Table 1. There was no difference between two groups in terms of age, FSH level on cycle day 3 and IVF cycle number. However, duration of infertility years, estradiol level on cycle day 3 showed significant difference. Treatment duration and total gonadotropin dose used were significantly higher in women undergoing the long GnRHa regimen compared with the GnRH-ant regimen (p<0.001 for both). Oocyte and embryo parameters after stimulation are given in Table 2. The number of oocytes retrieved, mature oocytes and fertilized oocytes were similar between two groups. Pregnancy rates were assessed in three groups as pregnancy, clinical pregnancy and early pregnancy loss rate and compared between two groups (Table 3). Clinical pregnancy rate was higher in agonist group but there was not a statistical difference between two groups (p=0.293). No difference was observed in early pregnancy loss rate between the groups. Good embryo quality rate was higher in antagonist group and showed significant difference when comparing with agonist group (p<0.001). However, embryo transfer day and the number of transferred embryos were similar in two groups and there was no statistically difference (Table 4).

## DISCUSSION

In the present study, the number of total and mature oocytes retrieved, and fertilized oocytes were similar in the long GnRHa and the GnRH-ant regimens. Although there was not a difference in pregnancy rate, the rate of good quality embryos was found to be higher in GnRH-ant group than long GnRHa group.

This study demonstrated a higher gonadotropin consumption and longer duration of stimulation with the long GnRHa regimen compared to the GnRH-ant regimen. Previous randomized controlled trials (RCT) comparing the efficacy of these regimens showed conflicting results owing to definition of poor response^(6,7)^. In a RCT by Cheung et al.^(7,8)^, they couldn’t find any difference regarding duration of stimulation, consumption of gonadotrophins in long GnRHa and GnRH-ant regimens. However, other studies are in line with our findings^(8,9,10)^. Morever, a recent meta-analysis comparing the long GnRHa versus GnRH-ant regimens in poor responders revealed that the duration of stimulation and gonadotropin dosage were significantly lower in GnRH agonist regimens^(10,11)^. However, the difference in these parameters could not be observed when micro-dose flare up protocol compared with the GnRH-ant protocol. Taken together, competitive binding of GnRH-ant to pituitary GnRH receptors shortens the stimulation cycle and reducing the dose of gonadotropin. Downregulation by the long GnRHa protocol significantly increases the gonadotropin dosage required and prolongs the stimulation period.

Our findings showed that there was no significant difference on the number of mature oocytes retrieved between the GnRH-ant and the long GnRH-a protocols, which was similar to the results of previous studies^(7,8,11,12)^. However, other studies demonstrated higher number of total oocyte retrieved in GnRHa regimen than GnRH-ant regimen^(12,13,14)^. Although the number of total oocytes retrieved was lower in the GnRH-ant protocol than the GnRHa protocol in these studies, number of mature oocytes retrieved was found to be similar. The rate of good quality embryos was higher in GnRH-ant than the long agonist regimen in our study. In contrast to our findings, Prapas et al. reported that mean embryo quality was significantly higher in the agonist group^(12,13)^. Although the rate of good quality embryos was higher in GnRH-ant group we found slightly lower clinical pregnancy rate (non-significant) in these women. This finding may be related to ill effect of GnRH-ant on endometrial receptivity. Rackow et al demonstrated that the use of GnRH-ant in comparison to GnRH-a in IVF cycles may be associated with impaired endometrial receptivity^(15)^. Moreover, a recent study comparing GnRHa and GnRH-ant regimens with the transfer of one high-quality embryo revealed that the GnRH-a group showed significantly higher pregnancy rate^(16)^. We are of the opinion that the reason for slightly lower clinical pregnancy rates in GnRH-ant IVF cycles in spite of higher quality embryos may be due to the decreased receptivity of the endometrium.

In our study, both groups presented a similar mean number of transferred embryos, and comparable pregnancy rate per transfer. Many randomized controlled trial (RCT) studies showed the similar results^(14,15,16,17,18,19,20)^. Additionally, no difference could be detected in early pregnancy loss rate between the groups in this study. Although early pregnancy loss rate was not compared between agonist and antagonist regimen in previous studies one retrospective study reported no significant difference in early pregnancy loss rate between poor and normo-responder women undergoing IVF^(18,21)^.

In conclusion, although there was no difference in pregnancy rates between the groups, lower gonadotropin consumption and shorter duration of ovarian stimulation in GnRH-ant cycles compared with the GnRHa cycles seem to decrease the cost and to make GnRH-ant more patient-friendly regimen for poor responders.

## Figures and Tables

**Table 1:**
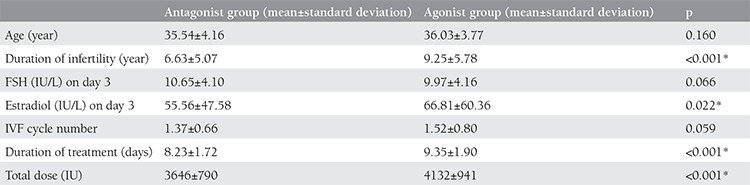
Demographic and clinical characteristics of patients

**Table 2:**
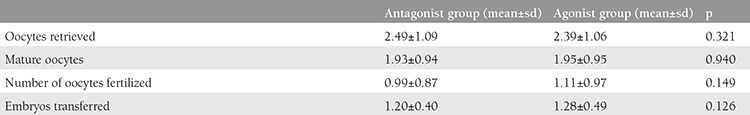
Oocyte and embryo parameters after stimulation in the groups

**Table 3:**
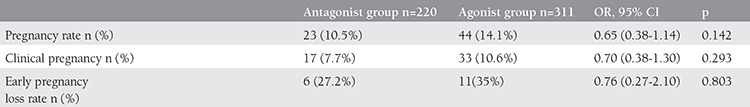
Comparison of pregnancy rates in two groups

**Table 4:**
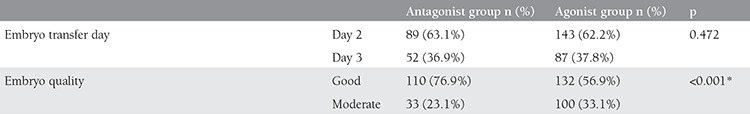
Comparison of two groups in terms of embryo transfer day and embryo quality
